# Endovascular treatment of multiple tuberculous mycotic aneurysm: A case report

**DOI:** 10.1097/MD.0000000000015268

**Published:** 2019-04-26

**Authors:** Shenyu Zhao, Zhe Wang, Yong Li, Hong Wang, Yu Zhao

**Affiliations:** a Department of Vascular Surgery, First Affiliated Hospital of Chongqing Medical University; b Department of Respiratory Medicine, People's Hospital of Tongliang District; c Academy of Life Sciences of Chongqing Medical University; d Yuanjiagang, Yuzhong District, Chongqing, China.

**Keywords:** endovascular treatment, hemodynamics, mycotic aneurysm, tuberculosis

## Abstract

**Rationale:**

We present a rare case of multiple tuberculous mycotic aneurysm. Multiple aneurysms caused by tuberculosis (TB) are difficult to treat. Here, we discuss a treatment modality using a microcore stent graft.

**Patient concerns:**

A 73-year-old man with pain in the back and on the right side of the chest associated with dry cough, presented with an inability to walk since 1-month.

**Diagnoses:**

A diagnosis of multiple aneurysms caused by TB was made, based on computed tomography (CT) scan and positive T-spot and Xpert tests.

**Interventions:**

We administered the empirical anti-TB regimen (pyrazinamide, isoniazid, rifampicin, and ethambutol) and performed endovascular repair using microcore stent graft.

**Outcomes:**

The post-operative hemodynamic analysis indicated that the patient's aneurysms no longer had a risk of rupture, and blood flow in the major branches of the aorta had been maintained. However, the patient could not survive due to a pulmonary infection acquired during recuperation at a local hospital.

**Lessons:**

For multiple tuberculous mycotic aneurysms, anti-TB therapy is inadequate and the microcore stent graft is a feasible option that can improve the hemodynamics in the aneurysms.

## 1 Introduction

Mycotic aneurysms are multiple aortic aneurysms formed due to infectious destruction of the vascular wall. The term was first coined by Osler in 1885.^[1]^ As the appearance of the deteriorated vessel resembled a fungal growth, Osler erroneously used the term mycotic aneurysm. Extension of the tuberculosis (TB) infection into the walls of the small pulmonary and meningeal arteries from the neighboring or contiguous inflammatory foci, are known to often cause aneurysms in the tuberculous cavities and meninges. However, tuberculous aneurysms of the large vessels, particularly the aorta are very rare. They account for 3% of all aneurysms of the abdominal aorta in necrotic preparations.^[2]^ The first case of tuberculous involvement of the aorta (aortitis) was reported in 1882 by Weigert, and the first case of tuberculous mycotic aneurysm of the aorta dates back to 1895.^[1]^ Since then, few cases have been published as case reports. No patients with a tuberculous aortic aneurysm (TBAA) are known to have survived, until the availability of combined technologies of modern imaging, anti-TB therapy, and vascular grafts. Miliary TB is a potentially fatal form of TB arising from the diffuse hematogenous spread of *Mycobacterium tuberculosis* to various parts of the body. Its radiographic imaging of the lungs is typical and is characterized by the appearance of multiple nodules a few millimeters in diameter (mm), in all lung fields.^[3]^ In this paper, we describe an interesting case of an elderly patient with mycotic aortic aneurysms, who had miliary TB with several complications.

This study was approved by the Ethical Committee of The First Affiliated Hospital of Chongqing Medical University.

## 2 Case report

In December 2017, a 73-year-old man was admitted with a 2-month history of pain in the back and right side of the chest associated with dry cough, and inability to walk for the last 1 month. He had a 50-year history of smoking and a 10-year history of chronic obstructive pulmonary disease (COPD). He was diagnosed with pulmonary TB based on positive T-spot and Xpert tests and computed tomography (CT)-scans at a local hospital 1 month ago. He was administered anti-TB treatment for 1month, comprising the empirical therapy using pyrazinamide, isoniazid, rifampicin, and ethambutol. Recent signs were weight loss and severe asthenia.

**Figure 1:**
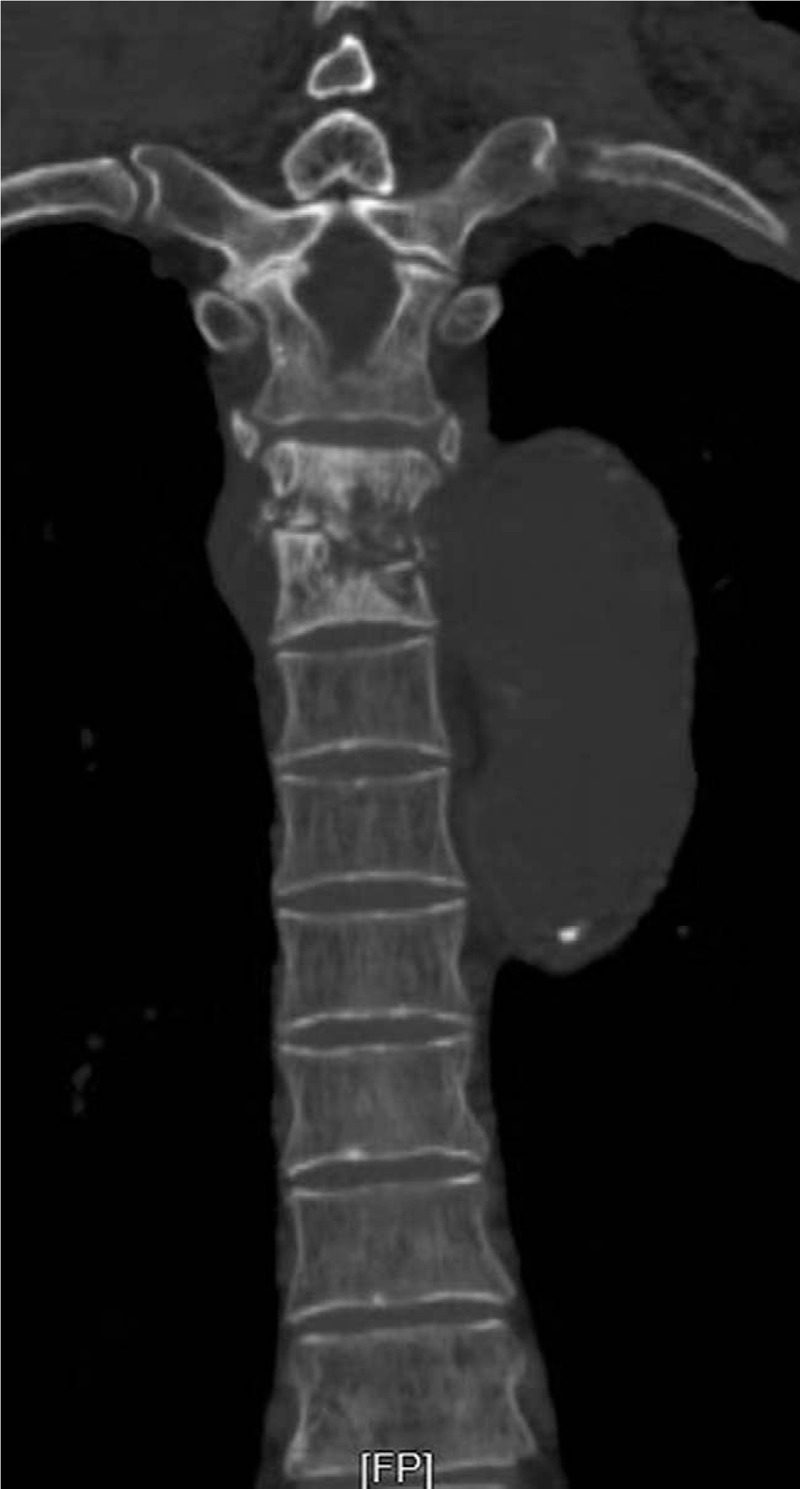
Chest CT showing osteolytic destruction at the 5-6th bodies of thoracic vertebrae and the saccular pseudoaneurysm in descending thoracic aorta. CT = computed tomography.

**Figure 2:**
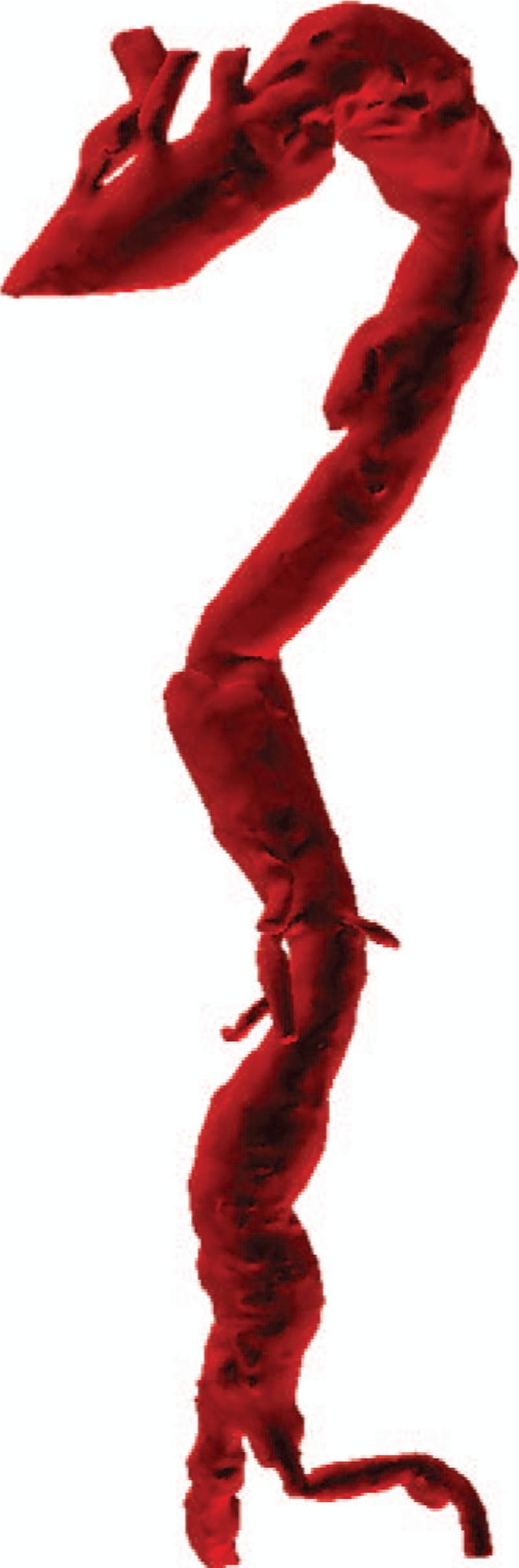
3D-computed tomography angiography showing the thoracic aortic aneurysm in aortic arch, the saccular pseudoaneurysm in descending thoracic aorta, another saccular pseudoaneurysm in descending thoracic aorta above celiac trunk, the abdominal aortic aneurysm and the right iliac aneurysm.

To investigate the patient's pain, a chest CT scan was performed, which revealed significant osteolytic destruction at the bodies of the 5th and 6th thoracic vertebrae and thoracic pedicles, with swelling of the soft tissue (Fig. 1). It was suspected that the destruction and the swelling of soft tissue were due to TB with cold abscess, resulting in paraplegia. The chest scan showed tiny, wide spread, and discrete pulmonary opacities with bilateral pleural effusion, and a lump in the lower lobe of the left lung which was suspected to be an inflammatory granuloma. There was 1 thoracic aortic aneurysm (116 mm × 40 mm) at the aortic arch, 1 saccular pseudoaneurysm (67 mm × 32 mm) in the descending thoracic aorta, another saccular pseudoaneurysm (69 mm × 34 mm) in the descending thoracic aorta above celiac trunk, 1 abdominal aortic aneurysm (112 mm × 33 mm) and 1 right iliac aneurysm (58 mm × 16 mm) (Fig. 2). A diagnosis of multiple tuberculous mycotic aneurysms of the aorta was made. Considering the patient's condition, surgery was not attempted. He underwent endovascular repair using microcore stent graft at our hospital. The surgical procedures are described below. The femoral artery was incised, and a microcore stent was implanted into the diseased artery. The stent covered a region ranging from the proximal normal aortic docking site to the site above the celiac trunk. The stent was not allowed to cover the openings of the major branches of the abdominal aorta, such as the celiac trunk, superior mesenteric artery, and bilateral renal arteries. Since the aneurysm involved important branches of the abdominal aorta, provisional observation was necessary. The procedures were performed under general anesthesia. Hemodynamic imaging of the distribution of wall pressure of blood flow in the aorta using computational fluid dynamics (CFD), was obtained before and after surgery in accordance with the preoperative and postoperative CT images in the digital imaging and communications in medicine (DICOM) format. On post-operative day 14, the patient was discharged without complications. The patient was in a stable condition and was transferred to a local hospital for continuation of the treatment of pulmonary infection and TB. Following transfer to the local hospital, the respiratory function deteriorated due to aggravated pulmonary infection. The patient was admitted to the Intensive Care Unit for type 1 respiratory failure and pulmonary infection. However, he could not survive the infection.

## 3 Discussion

An aortic aneurysm secondary to TB is rare. Reviews from both, pre- and post-antibiotic era, suggest the same.^[4,5]^ In recent times, most of the TBAA case reports have been from the industrialized world, where the prevalence of TB is very low. Mycobacteria might reach the aortic wall in 3 ways:

(1) by direct inoculation of the internal surface,
(2) from the vasa vasorum to the adventitia, and
(3) from a contiguous focus of infection.

Our patient developed thoracic TB of the bodies of 5th and 6th thoracic vertebrae, which lead to saccular pseudoaneurysm in the descending thoracic aorta through direct invasion. The other aneurysms were likely formed by direct inoculation of the blood vessel surface. The conventional treatment for a tuberculous aneurysm of the thoracic aorta is anti-TB chemotherapy combined with surgery, which involves extensive excision and debridement of the infected field, with in situ prosthetics.^[6,7]^ Although the reports of open-chest surgical series are satisfactory in terms of morbidity and mortality, in the patients with a challenging clinical presentation, such as acute hemorrhage, age over 70 years, malnutrition, weight loss, and bad respiratory function, a less invasive endovascular option is sometimes necessary.^[8,9]^ There is little research about the endovascular treatment of mycotic pseudoaneurysms; most of the literature details cases where a stent graft was initially deployed as a bridge to a definitive revascularization procedure.^[10,11]^ The major challenge with the endovascular approach is the impossibility of performing clean-up of the infected field. However, for patients with multiple aortic aneurysms, endovascular treatment is a better option.^[10]^

Thoracic endovascular aortic repair has emerged as an accepted alternative to surgery for the treatment of thoracic aortic aneurysm.^[12]^ This strategy is suitable for most patients, especially for those with poor physical condition because it is a minimally invasive procedure with lower perioperative complications and mortality rates.

**Figure 3:**
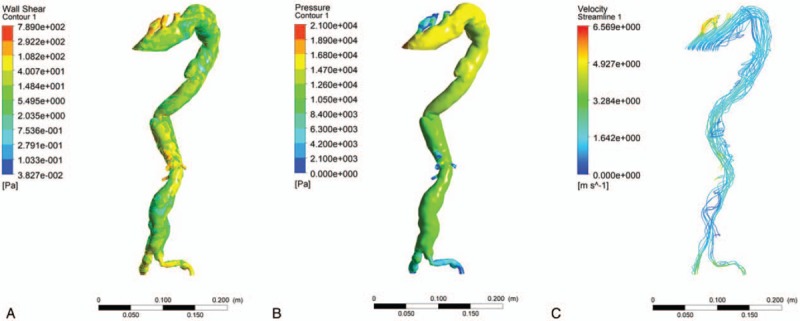
Hemodynamic analysis showing the pressure at the vessel wall (A), the wall shear stress (B) and the flowing structure of bloodstream(C) before surgery.

Our patient underwent 3D-computed tomography angiography (3D-CTA) of the entire aorta, and a hemodynamic analysis of the cardiac cycle using an individual-based model, and a surgical feasibility analysis was performed. Based on the results and the patient's history of past illness, we confirmed the diagnosis and the disease classification. During the preoperative hemodynamic analysis of the cardiac cycle, we found several high-risk factors in the patient:

1) high pressure on the vascular wall (Fig. 3A),
2) high wall shear stress (Fig. 3B),
3) turbulent flow of the bloodstream (Fig. 3C).

These indicated that the possibility of rupture of the aortic aneurysms was very high.^[13]^ Hence, we decided to perform an endovascular aortic repair.

We repaired the thoracic aneurysm in the aortic arch, the saccular pseudoaneurysm in the descending thoracic aorta, and another saccular pseudoaneurysm in the descending thoracic aorta above the celiac trunk, using the microcore stent graft. Considering the patient's condition, a hybrid surgery was not attempted. Traditional endovascular repair allows the stent graft to cover the openings of major branches of the aortic arch, such as the brachiocephalic trunk, common carotid artery, and subclavian artery. The microcore stent graft ensured blood supply to the important organs. For the abdominal aortic aneurysm and the right iliac aneurysm, we performed secondary endovascular repair based on the results of the first procedure. Since the patient's condition contraindicated a prolonged surgery.

**Figure 4:**
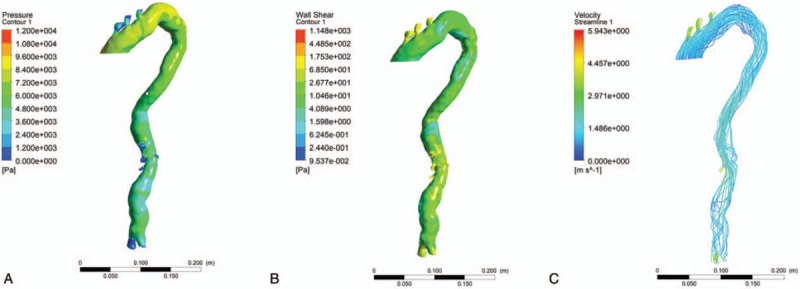
Hemodynamic analysis showing the pressure at the vessel wall (A), the wall shear stress (B) and the flowing structure of bloodstream(C) after surgery.

The patient underwent repeat 3D-CTAfor evaluation of his current hemodynamic status. Compared to the preoperative hemodynamic analysis, the following changes were observed:

1) lower pressure on the vascular wall (Fig. 4A),
2) lower wall shear stress (Fig. 4B),
3) the turbulent flow of the bloodstream had changed laminar flow (Fig. 4C).

**Figure 5:**
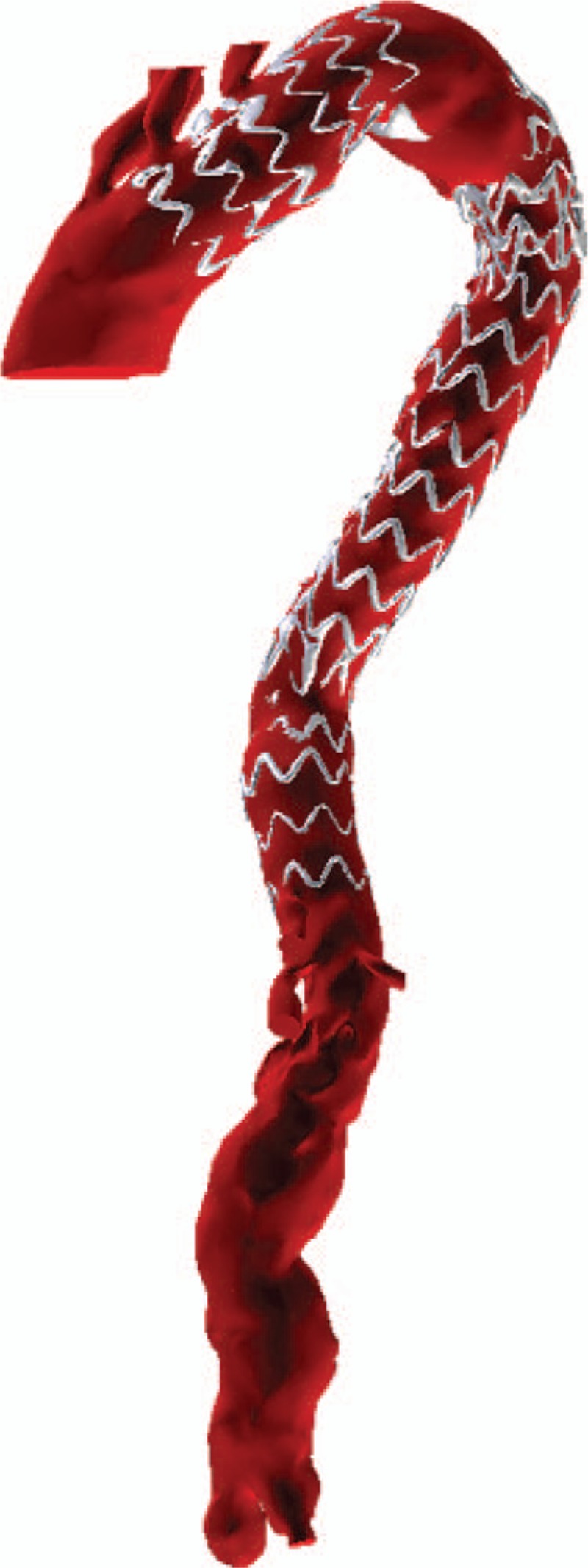
3D-computed tomography angiography showing the location of the stent graft and the aneurysm.

On 3D-CTA after surgery (Fig. 5), we found that the stent covered the openings of the common carotid and subclavian arteries; however, on hemodynamic analysis blood-supply of common carotid artery and subclavian artery was found to be normal, which confirmed that the stent did not reduce the blood supply to the organs. The blood flowed into the branches through the microcore stent graft. A comparison between the preoperative hemodynamic analysis and the postoperative hemodynamic analysis showed that in the aneurysm the wall shear stress and the rate of blood flow became lower and the flowing structure of the bloodstream had been changed to laminar flow which was the normal flowing structure. On postoperative CT, we observed that the stent graft was likely to promote the formation of blood clots in the aneurysms which would prevent their rupture. Based on the hemodynamic analysis, we found that the microcore stent has the following advantages: It maintains the blood flow in the important branch arteries and changes the flow from turbulent to laminar, by altering the hemodynamics. These changes reduce the blood flow velocity through the microcores of the stent, thereby reducing the shear stress on the wall of the sac and promoting thrombosis in the aneurysm sac.

This case report shows that endovascular treatment is effective and safe. Although the patient did not survive, the death was caused by a pulmonary infection. The postoperative hemodynamics showed that the condition of the blood-stream was normal. Thus, endovascular treatment using microcore stent graft for mycotic aneurysms, caused by TB, can be considered as a treatment option. For the population, who have a high incidence rate of TB, timely diagnosis and treatment are essential to reduce the rate of fatal complications such as tuberculous mycotic aneurysm.^[14]^

## Author contributions

**Data curation:** Yong Li, Yu Zhao.

**Investigation:** Shenyu Zhao, Yu Zhao.

**Methodology:** Shenyu Zhao, Zhe Wang, Yu Zhao.

**Project administration:** Shenyu Zhao.

**Resources:** Yong Li, Yu Zhao.

**Software:** Zhe Wang.

**Supervision:** Yu Zhao.

**Validation:** Zhe Wang.

**Visualization:** Zhe Wang, Hong Wang.

**Writing – original draft:** Shenyu Zhao.

**Writing – review & editing:** Shenyu Zhao.
